# The Willingness of Doctors to Adopt Artificial Intelligence–Driven Clinical Decision Support Systems at Different Hospitals in China: Fuzzy Set Qualitative Comparative Analysis of Survey Data

**DOI:** 10.2196/62768

**Published:** 2025-01-07

**Authors:** Zhongguang Yu, Ning Hu, Qiuyi Zhao, Xiang Hu, Cunbo Jia, Chunyu Zhang, Bing Liu, Yanping Li

**Affiliations:** 1 Economics and Management School Wuhan University Wuhan China; 2 Respiratory Centre China-Japan Friendship Hospital Beijing China; 3 School of Management Beijing University of Chinese Medicine Beijing China; 4 The Fourth Affiliated Hospital of Soochow University (Suzhou Dushu Lake Hospital) Suzhou China; 5 Business School Hubei University Wuhan China; 6 Hospital Office China-Japan Friendship Hospital Beijing China; 7 Department of Human Resources China-Japan Friendship Hospital Beijing China

**Keywords:** artificial intelligence, clinical decision support systems, willingness, technology adoption, fuzzy set qualitative comparative analysis, fsQCA, pathways

## Abstract

**Background:**

Artificial intelligence–driven clinical decision support systems (AI-CDSSs) are pivotal tools for doctors to improve diagnostic and treatment processes, as well as improve the efficiency and quality of health care services. However, not all doctors trust artificial intelligence (AI) technology, and many remain skeptical and unwilling to adopt these systems.

**Objective:**

This study aimed to explore in depth the factors influencing doctors’ willingness to adopt AI-CDSSs and assess the causal relationships among these factors to gain a better understanding for promoting the clinical application and widespread implementation of these systems.

**Methods:**

Based on the unified theory of acceptance and use of technology (UTAUT) and the technology-organization-environment (TOE) framework, we have proposed and designed a framework for doctors’ willingness to adopt AI-CDSSs. We conducted a nationwide questionnaire survey in China and performed fuzzy set qualitative comparative analysis to explore the willingness of doctors to adopt AI-CDSSs in different types of medical institutions and assess the factors influencing their willingness.

**Results:**

The survey was administered to doctors working in tertiary hospitals and primary/secondary hospitals across China. We received 450 valid responses out of 578 questionnaires distributed, indicating a robust response rate of 77.9%. Our analysis of the influencing factors and adoption pathways revealed that doctors in tertiary hospitals exhibited 6 distinct pathways for AI-CDSS adoption, which were centered on technology-driven pathways, individual-driven pathways, and technology-individual dual-driven pathways. Doctors in primary/secondary hospitals demonstrated 3 adoption pathways, which were centered on technology-individual and organization-individual dual-driven pathways. There were commonalities in the factors influencing adoption across different medical institutions, such as the positive perception of AI technology’s utility and individual readiness to try new technologies. There were also variations in the influence of facilitating conditions among doctors at different medical institutions, especially primary/secondary hospitals.

**Conclusions:**

From the perspective of the 6 pathways for doctors at tertiary hospitals and the 3 pathways for doctors at primary/secondary hospitals, performance expectancy and personal innovativeness were 2 indispensable and core conditions in the pathways to achieving favorable willingness to adopt AI-CDSSs.

## Introduction

### Background

Artificial intelligence–driven clinical decision support systems (AI-CDSSs), aimed at enhancing medical decision-making, have been in use since the 1980s [1]. These systems leverage clinical knowledge, patient information, and health data to improve health care services by allowing intelligent detection, management, and improvement of patient health conditions [2,3]. According to statistics, the total number of outpatient visits to medical and health institutions in China reached 5.5 billion in 2023, with doctors at public hospitals averaging 7.1 outpatient visits and 2.3 hospital visits per day and with the burden on primary health care institutional doctors being even greater [4]. According to “World Health Statistics 2023: Monitoring Health for the SDGs, sustainable development goals” released by the World Health Organization (WHO), China has a low doctor density and uneven distribution of health care resources [5]. To relieve the pressure on doctors and improve the efficiency of medical services, China has actively explored the application of AI-CDSSs, with the National Health Commission of China issuing a national policy on the promotion of clinical decision support systems (CDSSs) in 2023.

With the evolution of artificial intelligence (AI) technology, current AI-CDSSs can generate specific assessments and recommendations through logical reasoning, analyze patient data from electronic medical records, and offer decision support to health care providers [6]. AI-CDSSs are widely used in clinical diagnoses, drug treatments, preventive measures, and patient management, and are recognized for their roles in enhancing health care decision-making and doctor performance [7]. For instance, scholars, such as Hanson et al [8], have conducted research from the perspective of patient management, evaluating the application of AI-CDSSs in the nursing field, and have confirmed that certain systems are able to improve the accuracy and comprehensiveness of nursing treatment. Alsharqi et al [9], among others, have evaluated the application effects of AI-CDSSs in the automatic image selection field of echocardiography. They found that AI-CDSSs can effectively identify, distinguish, and explain images through machine learning models. Islam et al [10] studied how AI-CDSSs could help patients continuously observe different parameters for controlling insulin levels, automatically analyzing the personal data of diabetic patients. The application of AI-CDSSs in the field of anesthesiology is also extensive. According to existing research, AI-CDSSs can improve the preoperative use of antibiotics and beta-blockers, reduce the use of inhaled anesthetics, and assist in completing anesthesia records and billing work [11-13].

AI-CDSSs are primarily targeted at physicians, whose acceptance is pivotal for their implementation. Sambasivan et al [14] extended the unified theory of acceptance and use of technology (UTAUT) model and explored the factors influencing doctors’ willingness to adopt new CDSSs in a developing country context, considering user involvement in decision-making, perceived threat to autonomy, effort expectancy, and performance expectancy. Their study involved doctors from 12 hospitals representing 10 different specialty areas. However, physicians’ attitudes toward these systems varied, with some exhibiting favorable inclinations and others exhibiting reluctance, primarily attributed to apprehensions about technological readiness, data confidentiality, absence of personalized interaction, and skepticism toward emerging technologies.

Thus, although some doctors have expressed positive attitudes toward adopting AI-CDSSs, others still harbor reservations, citing concerns over technology immaturity, data privacy and security, absence of human touch, and a distrust of new technologies. For instance, Wagner et al [15] found that only 54.9% of family doctors were willing to use AI for medical diagnosis, while O'Leary et al [16] reported that 82% of health care professionals saw the usefulness of AI in diagnosing rare diseases. In their 2020 study, Park et al [17] discovered that over 75% of American radiology students believed AI would play a crucial role in future medicine, while almost half of them expressed decreased enthusiasm for radiology due to AI adoption. Scheetz et al [18] found that 71% of the health care professionals surveyed in Australia and New Zealand believed AI would improve medicine, while 85.7% felt it would impact health care manpower. In a South Korean study, only 5.9% of doctors were very familiar with AI [19]. Similarly, in a UK study, medical students indicated they were not adequately prepared for AI. Scholars have also expressed concerns about humans being replaced by AI in health care [20].

Thus, despite the potential benefits, skepticism persists among scholars and doctors regarding the integration of AI in health care, citing concerns about technology replacing human roles. For instance, Poon and Sung [21] highlighted doctors’ skepticism toward AI technology in clinical practice that impeded the progress of AI applications owing to a lack of trust.

Investigating doctors’ willingness to adopt AI-CDSSs and understanding the factors influencing their acceptance can have a significant impact on the comprehensive integration of AI-CDSSs into clinical applications. Sambasivan et al [14] employed structural equation modeling to explore doctors’ willingness to use AI-CDSSs in developing countries, revealing that concerns about potential threats to professional autonomy could dampen doctors’ willingness to embrace these systems. Conversely, active involvement in the planning, design, and implementation of AI-CDSSs was associated with increased acceptance and readiness to use these technologies. Similarly, Laka et al [22] utilized logistic regression analysis to explore the adoption willingness of AI-CDSSs, finding that doctors in local primary care facilities, as opposed to those in larger hospitals, identified factors, such as time constraints, perceived threats to professional autonomy, and considerations of patient preferences, as significant barriers to adopting AI-CDSSs.

Current research has focused mainly on examining the impact of individual factors or certain factors affecting the allocation of medical resources. Thus, the literature lacks in-depth analysis of the interactive mechanisms and synergistic effects of factors influencing doctors’ intentions to adopt AI-CDSSs. There is also a lack of comprehensive analysis that combines the multiple factors influencing doctors’ intentions to adopt AI-CDSSs for assessing their causal relationships. To explore the driving mechanisms of multiple conditional linkages on doctors’ adoption willingness, our study focuses on doctors at different medical institutions in China, not only providing important assistance for the application of AI-CDSSs in hospitals at all levels in China, but also offering experiential references for other developing countries in the use and adoption of AI-CDSSs. Using fuzzy set qualitative comparative analysis (fsQCA), we clarify the synergistic effects of multiple factors influencing doctors’ intentions to adopt AI-CDSSs, thereby providing theoretical support for promoting their application in the medical field.

### Framework for AI-CDSS Adoption Willingness

The integration of the UTAUT was proposed in 2003 by Venkatesh et al [23] to explain the relevant factors influencing an individual’s willingness to accept or use new technology. The UTAUT consists of 4 key factors: performance expectancy, effort expectancy, social influence, and facilitating conditions, which influence behavior through willingness [24]. The UTAUT also considers the moderating effects of sex, age, experience, and voluntary use. The theory was established in the context of the organizational implementation of new technology, with the influencing factors having clear utilitarian characteristics. With the emergence of AI technology, an increasing number of scholars are using the UTAUT to study individual AI technology adoption issues. The technology-organization-environment (TOE) framework proposed by Tornatzky et al [25] suggests that technological, organizational, and external environmental factors also have a certain impact on an organization’s adoption and implementation of new technology.

There is an interactive relationship between medical institutions and doctors. An organization’s attitude toward innovation will affect employees’ acceptance of new technology [26]. In addition, the adoption willingness and behavior of AI technology in an organization may be affected by the adoption willingness of employees [27]. Therefore, based on the UTAUT model and TOE framework, this study constructed a multi-layer dynamic impact model for AI technology adoption among physicians.

### Technical Factors Influencing the Adoption of AI-CDSSs by Doctors

Performance expectancy is one of the key constructs in the UTAUT model used to explain and predict individual technology acceptance behavior [28]. In a hospital setting, it can capture the extent that doctors believe that using new technology will help improve their job performance [29]. Previous studies have shown that performance expectancy is crucial for doctors’ adoption and acceptance of AI-CDSSs [30], similar to the perceived usefulness in the technology acceptance model (TAM). Compared with other technology application scenarios, doctors place more emphasis on the impact of technology on their job performance when adopting new technology. Currently, AI technology has the ability to assist in eliminating redundant work steps, providing decision support, and improving job performance [31,32]. However, there are also issues, such as communication barriers between doctors and AI technology, which can impact work efficiency. The effectiveness of AI technology in the workplace is yet to be widely validated [33]. Therefore, performance expectancy still plays an important role in doctors’ willingness to adopt AI technology.

Perceived risk refers to the degree of insecurity that doctors perceive when they are using technology to execute tasks and exchange data [34]. AI technology requires big data to achieve powerful learning, which means that AI technology may involve the input of data from various parties, such as individuals and vendors. When there is a risk of information leakage, doctors using AI technology may face legal, moral, and ethical issues. This can have a significant impact on their willingness to adopt AI technology.

### Organizational Factors Influencing the Adoption of AI-CDSSs by Doctors

Social influence refers to the influence doctors feel from their social environment regarding a specific behavior. It is a key factor in the UTAUT model that affects an individual’s willingness to adopt new technology [30]. In an organizational context, doctors are frequently influenced by colleagues and leaders, and they enhance their sense of belonging by conforming to these groups. When faced with emerging technologies, such as AI, there may not be enough information for an informed decision, making doctors more susceptible to peer influence. However, leaders also influence doctors’ willingness to adopt AI technology [35] and have the power to authorize subordinates, determine job promotions, provide rewards, and administer punishments. Thus, doctors align with leaders to receive recognition.

The UTAUT construct “facilitating conditions” refers to the extent that doctors perceive that the necessary infrastructure and resources in the organization support their use of new technology [30]. Thus, this would also influence a doctor’s willingness to adopt AI technology. The promotion of new technology requires organizations to provide various resources such as knowledge, funds, and technology. Simpler and more convenient external support conditions are associated with a greater likelihood of doctors adopting AI technology. Research has shown that facilitating conditions positively influence an individual’s willingness to adopt AI technology [36,37].

### Individual Factors Influencing the Adoption of AI-CDSSs by Doctors

Technology anxiety refers to an individual’s emotional anxiety or fear of the performance of new technology. For example, when individuals believe that the technology may threaten their sense of self, they may experience technology anxiety, which reduces their willingness to adopt it [28]. The existing and potential capabilities of AI technology to replace human abilities are constantly increasing, causing individuals to experience stronger feelings of anxiety compared with other technologies. Therefore, our study included technology anxiety as a factor in our framework.

Personal innovativeness reflects an individual’s willingness to try something new. Innovation diffusion theory suggests that owing to differences in innovation capabilities, individuals’ willingness and behaviors vary in this respect. Some scholars have proven that in consumer scenarios, personal innovativeness positively influences individuals’ willingness to adopt self-service technologies [38]. As AI technology is a revolutionary innovation, personal innovativeness is needed to drive doctors toward a greater willingness to adopt it. Therefore, we assume that personal innovativeness significantly impacts doctors’ willingness to adopt AI technology.

### Study Model

Our study applies the UTAUT model for the basis of our research framework to analyze the factors influencing doctors’ adoption of AI-CDSSs, adding other factors as well. Our model considers 3 perspectives: technology, organizations, and individuals. The technical factors included are performance expectancy and perceived risk, the organizational factors are social influence and facilitating conditions, and the individual factors are technology anxiety and personal innovativeness. We have incorporated performance expectancy, social influence, and facilitating conditions from the UTAUT model, and based on the research by Chen et al [37], personal innovativeness and perceived risk have been introduced into the model and confirmed, along with key factors in the UTAUT model that significantly influence doctors’ acceptance of AI technology. Huang et al [39] demonstrated how technology anxiety reflected an individual’s emotional anxiety or fear regarding the performance of AI technology. When individuals perceived the technology as threatening their sense of self, they experienced technology anxiety, which reduced their willingness to adopt the technology. Therefore, we have included technology anxiety in the model. Considering that AI technology often does not require users to learn how to operate it, as it possesses anthropomorphic characteristics that are different from nonintelligent technologies [8], we did not include effort expectancy. The factors ultimately in our analysis framework are performance expectancy, perceived risk, facilitating conditions, social influence, technology anxiety, and personal innovativeness.

**Figure 1:**
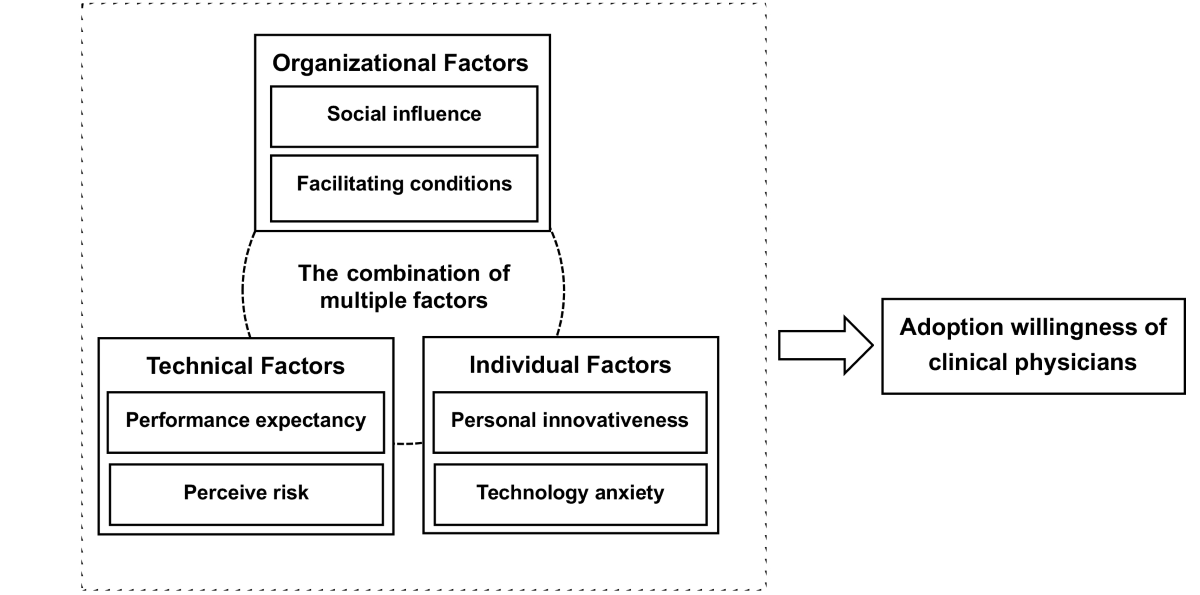
Analysis framework of the factors influencing Chinese doctors’ willingness to adopt artificial intelligence–driven clinical decision support systems.

Thus, we assume that doctors’ willingness to adopt AI technology will be influenced by these various factors. Although some studies have investigated the individual effects of these elements on doctors’ AI adoption willingness, providing a foundation for our understanding of the factors influencing the willingness to adopt AI technology, research struggles to answer how these factors interact to influence the willingness to adopt AI technology under multiple situational conditions. Additionally, research has not identified the deep-rooted causal relationships that are affecting doctors’ willingness to adopt AI technology. To fill this gap in the literature, based on the UTAUT model and incorporating the TOE framework, we have explored the complex causal mechanisms of how environmental, technological, and individual factors influence doctors’ willingness to adopt AI technology from a configurational perspective, proposing a theoretical model (Figure 1).

## Methods

### Method Selection

We used fsQCA to explore the complex causal mechanisms influencing clinical doctors’ willingness to adopt AI-CDSSs, primarily for the following reasons. First, using this method uncovers the nonlinear relationships between various influencing factors and the doctors’ willingness to adopt AI-CDSSs. The fsQCA method also explores combinations of influencing factors instead of individual factors [40]. Second, as our research question was “Which factors can lead clinical doctors to have a higher willingness to adopt AI-CDSSs?”, the use of this method can reveal multiple equivalent paths that influence doctors’ willingness to adopt AI-CDSSs. Third, compared with other qualitative comparative analysis methods, fsQCA is more suitable for handling continuous variables. Fourth, fsQCA can be applied to different sample sizes ranging from very small (<50 cases) to very large (thousands of cases) [41].

### Data Collection

An online questionnaire was developed and shared with doctors in China who met specific criteria related to their work experience. We distributed 578 questionnaires through the Wenjuanxing platform [42]. After eliminating any invalid submissions, we had 450 valid responses, resulting in a questionnaire response rate of 77.9%. Detailed demographic information on the participants is presented in Table 1.

According to “Hospital Classification Management Measures” issued by the National Health Department, hospitals in China are classified into 3 levels. Tertiary hospitals provide medical and health services across regions, provinces, and cities, and nationwide. Secondary hospitals provide comprehensive medical and health services to multiple communities and undertake teaching and research tasks in regional hospitals. Primary hospitals are grassroot hospitals and health centers that provide preventive, medical, health, and rehabilitation services to their local communities [43]. In this study, we divided hospitals into only 2 categories for simplicity: tertiary hospitals and primary/secondary hospitals. Among the respondents, there were 332 responses from clinical doctors working in tertiary medical institutions and 118 responses from clinical doctors in primary/secondary medical institutions, as mentioned above.

**Table 1:** Basic respondent demographic statistics.

Category			Tertiary hospitals (N=332), n (%)		Primary/secondary hospitals (N=118), n (%)
**Sex**					
	Male	171 (51.5)		53 (44.9)	
	Female	161 (48.5)		65 (55.1)	
**Age (years)**					
	Under 25	27 (8.1)		12 (10.2)	
	25 to 34	109 (32.8)		31 (26.3)	
	35 to 44	142 (42.8)		35 (29.7)	
	45 to 54	51 (15.4)		34 (28.8)	
	Above 54	3 (0.9)		6 (5.1)	
**Education**					
	Bachelor’s degree	89 (26.8)		85 (72.0)	
	Master’s degree	176 (53.0)		13 (11.0)	
	Doctoral degree	66 (19.9)		1 (0.9)	
	Others	1 (0.3)		19 (16.1)	
**Major title**					
	Resident physician	100 (30.1)		46 (39.0)	
	Attending physician	113 (34.0)		39 (33.1)	
	Associate chief physician	81 (24.4)		19 (16.1)	
	Chief physician	38 (11.5)		14 (11.9)	
**Duration of employment (years)**					
	1 or less	28 (8.4)		15 (12.7)	
	2 to 5	86 (25.9)		25 (21.2)	
	6 to 10	71 (21.4)		17 (14.4)	
	11 to 15	66 (19.9)		23 (19.5)	
	16 to 20	41 (12.4)		5 (4.2)	
	21 to 25	19 (5.7)		11 (9.3)	
	25 or more	21 (6.3)		22 (18.6)	

### Variable Measurement and Calibration

To ensure the reliability and validity of our scales, we based them on mature scales developed by scholars in the field of technology adoption and appropriately adjusted them for our research question to create measurement items for our main variables [23]. The main variables were performance expectancy, perceived risk, facilitating conditions, social influence, technology anxiety, personal innovativeness, and adoption willingness. Participant responses were on a 5-point Likert scale assessing the extent that they agreed with the content described in the items. We calculated the average score of the corresponding items within each scale to measure the variable. We use SPSS (version 25.0) to analyze the reliability and validity of the scales. All variables had a Cronbach α greater than .8, composite reliability greater than 0.8, Kaiser-Meyer-Olkin (KMO) values greater than 0.7, and average variance extracted values greater than 0.5, indicating that the scales had good reliability and validity. To ensure the overall reliability of our results, we removed items with factor loadings less than 0.6, and the conditions were still met after these items were deleted (Table 2).

To meet the Boolean logic requirements for qualitative comparative analysis, variables need to be transformed into sets and cases need to be assigned to the sets before conducting fsQCA, a process known as data calibration. In this process, we need to establish calibration points for “full membership,” “crossing point,” and “full nonmembership.” We adopted a scholar’s calibration method for the Likert scale questionnaire data, coding “completely agrees (5)” as “full membership,” “neutral (3)” as “crossing point,” and “completely disagrees (1)” as “full nonmembership.” By setting these 3 thresholds, we converted the original data into fuzzy scores ranging from 0 to 1, using the calibrate (x, n1, n2, n3) function in the fsQCA software.

**Table 2:** Reliability and validity analysis.

Variable and item			Factor loading		Cronbach α		KMO^a^		CR^b^		AVE^c^
**Performance expectancy**					.831		0.797		0.916		0.611
	1	0.913									
	2	0.925									
	3	0.884									
	4	0.543									
**Perceived risk**					.879		0.848		0.934		0.568
	1	0.567									
	2	0.674									
	3	0.885									
	4	0.875									
	5	0.838									
**Facilitating conditions**					.857		0.821		0.929		0.640
	1	0.775									
	2	0.893									
	3	0.768									
	4	0.825									
**Social influence**					.883		0.811		0.940		0.864
	1	0.842									
	2	0.913									
	3	0.810									
	4	0.686									
**Technology anxiety**					.921		0.900		0.890		0.572
	1	0.839									
	2	0.833									
	3	0.859									
	4	0.620									
	5	0.723									
**Personal innovativeness**					.865		0.831		0.896		0.650
	1	0.786									
	2	0.824									
	3	0.912									
	4	0.880									
**Adoption willingness**					.887		0.747		0.931		0.768
	1	0.961									
	2	0.953									
	3	0.946									

^a^KMO: Kaiser-Meyer-Olkin.

^b^CR: composite reliability.

^c^AVE: average variance extracted.

### Ethical Considerations

This study was approved by the Clinical Research Ethics Committee of China-Japan Friendship Hospital (number: 2024-KY-254). All participants provided informed consent before the investigation began. Furthermore, information on the research participants was kept confidential, and personal private information was not disclosed.

## Results

### Necessary Condition

According to the fsQCA method, before conducting the configuration analysis, the first step is to perform a necessity analysis on the individual condition variables, with the results reflected through consistency and coverage. Consistency represents the degree that the condition variables are a subset of the outcome variables. Identifying a necessary condition generally requires a consistency score higher than 0.9. Coverage represents the extent that the condition variables explain the outcome. This is only meaningful for conditions that pass the consistency test, with no acceptable threshold. The results of the necessity test for the individual conditions are shown in Table 3.

In Table 3, we can see that the consistency of personal innovativeness in influencing doctors’ willingness to adopt AI-CDSSs at primary/secondary hospitals was higher than 0.9 and coverage was as high as 0.933. This indicates that personal innovativeness is a necessary condition influencing doctors’ willingness to adopt AI-CDSSs in these hospitals. The consistency of the other variables was less than 0.9, indicating that they are not sufficient to constitute the necessary conditions affecting doctors’ willingness to adopt AI-CDSSs, with these variables having relatively weak independent explanatory powers. Thus, we need to further analyze the combining effects of these condition variables and their impacts on our outcome variable.

**Table 3:** Necessary condition analysis.

Condition variable	Tertiary hospitals		Primary/secondary hospitals	
	Consistency	Coverage	Consistency	Coverage
Performance expectancy	0.882	0.917	0.872	0.832
~^a^Performance expectancy	0.376	0.852	0.423	0.784
Perceived risk	0.471	0.952	0.570	0.890
~Perceived risk	0.775	0.853	0.722	0.763
Facilitating conditions	0.653	0.955	0.757	0.924
~Facilitating conditions	0.611	0.849	0.567	0.739
Social influence	0.787	0.944	0.854	0.934
~Social influence	0.487	0.855	0.479	0.711
Technology anxiety	0.707	0.939	0.661	0.908
~Technology anxiety	0.555	0.854	0.658	0.766
Personal innovativeness	0.861^b^	0.949	0.930^b^	0.933
~Personal innovativeness	0.414	0.834	0.414	0.702

^a^“~” indicates the negation of the condition.

^b^Consistency exceeds 0.8.

### Adequacy Analysis of Configuration

As mentioned, we conducted fsQCA separately for tertiary and primary/secondary hospitals. According to the principles of fsQCA, we included 6 condition variables. We retained 85% of the case numbers to set the frequency threshold, with case number thresholds of 5 and 2 for tertiary hospitals and primary/secondary hospitals, respectively. The consistency threshold for each configuration was higher than 0.8, and the proportional reduction in inconsistency threshold was greater than 0.75. A configuration with consistency below the threshold was assigned a value of 0 (Tables 4 and 5).

After standard analysis of the improved truth table, we found 3 types of solutions: complex, intermediate, and parsimonious. Among them, we obtained the intermediate solution through a counterfactual analysis, assuming that the emergence of personal innovativeness may increase doctors’ willingness to adopt AI-CDSSs, whereas the other individual conditions may contribute to doctors’ willingness to adopt AI-CDSSs. We identified the core conditions for each configuration by comparing the nested relationships between the intermediate and parsimonious solutions. The conditions appearing in both the parsimonious and intermediate solutions were considered core conditions for that configuration, whereas those appearing only in the intermediate solution were considered marginal conditions (Table 6).

In Table 6, we can see that there are 3 pathways leading to positive doctors’ willingness to adopt AI-CDSSs in tertiary hospitals, which have been presented below.

**Table 4:** Truth table for doctors at tertiary hospitals.

Conditional variable							Number		Outcome		Raw consistency		PRI^a^ consistency		SYM^b^ consistency
A	B	C	D	E	F			Y							
1	1	1	1	1	1	32		1		0.997789		0.994404		0.994404	
1	1	0	1	1	1	7		1		0.997023		0.989236		0.989237	
1	0	1	1	1	1	38		1		0.995619		0.988805		0.988805	
1	1	1	1	0	1	5		1		0.996540		0.984514		0.987172	
1	0	0	1	1	1	22		1		0.994604		0.984282		0.984282	
1	0	1	1	0	1	44		1		0.992934		0.981120		0.981120	
1	0	0	0	1	1	19		1		0.992382		0.976033		0.978558	
1	0	0	1	0	1	10		1		0.990879		0.962754		0.963750	
1	0	0	0	1	0	8		1		0.988872		0.942290		0.942291	
0	0	1	1	0	1	5		1		0.988860		0.929057		0.932779	
1	0	0	0	0	1	13		1		0.982544		0.921498		0.931327	
1	0	0	1	0	0	8		1		0.984218		0.907492		0.907493	
0	0	0	0	1	1	8		1		0.981872		0.904679		0.909656	
0	0	0	1	0	1	6		1		0.985961		0.902044		0.902046	
1	0	1	1	0	0	10		1		0.971584		0.850015		0.866264	
0	0	0	0	0	1	10		1		0.973338		0.826399		0.828025	
1	0	0	0	0	0	11		1		0.964521		0.796661		0.797960	
0	0	0	0	0	0	24		0		0.895842		0.463338		0.467589	

^a^PRI: proportional reduction in inconsistency.

^b^SYM: symmetric.

**Table 5:** Truth table for doctors at primary/secondary hospitals.

Conditional variable							Number		Outcome		Raw consistency		PRI^a^ consistency		SYM^b^ consistency
A	B	C	D	E	F			Y							
1	0	1	1	1	1	7		1		0.995976		0.986577		0.986577	
1	1	1	1	1	1	15		1		0.991665		0.977517		0.977518	
1	0	0	1	1	1	2		1		0.992201		0.963961		0.963961	
1	1	0	1	1	1	3		1		0.988830		0.953054		0.953054	
1	1	0	1	0	1	3		1		0.992045		0.952049		0.952050	
1	0	1	1	0	1	17		1		0.982767		0.947533		0.953350	
0	0	1	1	0	1	2		1		0.989427		0.927492		0.927492	
1	1	1	1	0	1	4		1		0.984086		0.922251		0.948591	
1	0	0	1	0	1	3		1		0.986501		0.920241		0.920242	
1	0	1	0	0	1	3		1		0.975536		0.830054		0.830053	
1	0	0	0	0	1	5		1		0.969439		0.797216		0.797216	
0	0	0	1	0	1	2		0		0.968835		0.732559		0.732558	
0	0	0	0	0	1	6		0		0.946550		0.654310		0.654310	
1	0	1	1	0	0	4		0		0.965158		0.631557		0.631558	
0	0	0	0	1	0	2		0		0.912641		0.349602		0.349602	
0	0	0	0	0	0	16		0		0.842963		0.315754		0.327492	
1	1	0	0	0	0	3		0		0.937445		0.308522		0.308522	
0	1	0	0	1	0	2		0		0.937238		0.290340		0.348758	
1	0	0	0	0	0	10		0		0.861165		0.251138		0.251138	

^a^PRI: proportional reduction in inconsistency.

^b^SYM: symmetric.

**Table 6:** Adoption willingness of doctors in different medical institutions.

Conditions	Tertiary hospitals^a^							Primary/secondary hospitals^b^			
	Pathway S1		Pathway S2		Pathway S3			Pathway N1		Pathway N2	
	S1a	S1b	S2a	S2b	S3a	S3b			N2a		N2b
Performance expectancy	PC^c^	PC	—^d^	—	PC	PC	PC		PC		—
Perceived risk	AM^e^	AM	AM	AM	—	—	—		AM		AM
Facilitating conditions	AM	—	AM	—	PM^f^	—	—		—		PM
Social influence	AM	PM	AM	PM	PM	PM	PM				PM
Technology anxiety	—	AM	—	AM	—	PM	—		AM		AM
Personal innovativeness	—	—	PC	PC	PC	PC	PC		PC		PC
Consistency	0.963	0.970	0.964	0.985	0.992	0.994	0.972		0.961		0.983
Raw coverage	0.416	0.451	0.413	0.444	0.591	0.549	0.769		0.514		0.479
Unique coverage	0.018	0.015	0.017	0.011	0.022	0.040	0.279		0.023		0.017

^a^The solution consistency value was 0.804 and solution coverage value was 0.953.

^b^The solution consistency value was 0.810 and solution coverage value was 0.961.

^c^PC: presence of a core causal condition.

^d^The condition can or cannot exist in the configuration.

^e^AM: absence of a marginal causal condition.

^f^PM: presence of a marginal causal condition.

### Configuration for Doctors at Tertiary Hospitals

#### Technology Driven

The core condition for both pathways S1a and S1b was performance expectancy, which played a dominant role in the pathways. The findings indicate that these doctors believe that AI-CDSSs are helpful in clinical work, can improve work efficiency, and can enhance work quality. Pathway S1a indicated that under high performance expectancy, even without perceived technical risks for AI-CDSSs and without organizational factors, such as convenience of AI use and social influence, doctors have favorable adoption willingness for AI-CDSSs. Pathway S1b indicated that under high performance expectancy, without technology anxiety and perceived risk but with social influence, such as influence from surrounding groups, doctors still have favorable adoption willingness for AI-CDSSs.

#### Individual Driven

The core condition for pathways S2a and S2b was personal innovativeness, which played a primary role in these pathways. The findings indicate that these doctors are willing to try new AI technology, as they typically favor innovativeness that enables continuous learning of new medical technologies and treatment methods. Pathway S2a showed that when doctors reflect strong personal innovativeness, without perceived risks, convenience factors, and social influence, they have positive adoption willingness for AI-CDSSs. Pathway S2b indicated that when doctors have personal innovativeness without perceived risks and technology anxiety for AI-CDSSs, but with a certain degree of social influence, they still tend to have positive adoption willingness for AI-CDSSs.

#### Technology-Individual Dual Driven

The core conditions for pathways S3a and S3b were performance expectancy and personal innovativeness, indicating that doctors with both high performance expectancy and high personal innovativeness develop strong adoption willingness. Pathway S3a demonstrated that doctors with high performance expectancy and high personal innovativeness need support from certain convenience factors as well as a certain degree of social influence to engender strong adoption willingness for AI-CDSSs. Pathway S3b showed that when doctors have high performance expectancy, high personal innovativeness, and some degree of technology anxiety and social influence, they still tend to have high adoption willingness for AI-CDSSs.

### Configuration for Doctors at Primary and Secondary Hospitals

#### Technology-Individual Dual Driven

The core conditions for pathways N1 and N2a were performance expectancy and personal innovativeness, indicating that doctors with both high performance expectancy and high personal innovativeness have strong adoption willingness for AI-CDSSs. Pathway N1 showed that doctors with high performance expectancy and high personal innovativeness have strong adoption willingness when influenced by leaders, colleagues, and other people regarding the use of AI. Pathway N2a demonstrated that doctors with high performance expectancy, high personal innovativeness, and no perceived risks or technology anxiety for AI technology have strong adoption willingness.

#### Organization-Individual Dual Driven

The core conditions for pathway N2b were convenience factors and personal innovativeness, indicating that doctors who receive support from their workplace and from technology and have high personal innovativeness develop strong adoption willingness. In other words, with high convenience factors, personal innovativeness, and low social influence, even without perceived risks and technology anxiety, doctors have strong adoption willingness for AI-CDSSs.

### Comparative Analysis

Comparing doctors at tertiary hospitals with those at primary/secondary hospitals, we can point out the similarities and differences. The pathways reflect what drives the doctors at these different medical institutions to stronger adoption willingness for AI-CDSSs.

#### Similarities

AI technology and personal factors play dominant roles in influencing the adoption of AI-CDSSs by doctors at all the hospitals analyzed. There are not only single-dimensional factors that affect doctors’ adoption willingness, but also combinations of factors, as in the technology-individual dual model, that have an impact on adoption. At the technological level, doctors believed that the application of AI-CDSSs in clinical diagnosis and treatment processes can provide efficient diagnostic support and improve the quality of clinical services. Although issues, such as overdiagnosis, may exist with AI-CDSSs, doctors considered the overall technology of AI-CDSSs to be safe and reliable. At the individual level, doctors demonstrated strong acceptance and openness to AI technology, showing no anxiety regarding the emergence of new technologies. In particular, they appeared willing to try new AI technologies.

#### Differences

Convenience factors had a greater impact on the adoption willingness of doctors at primary/secondary hospitals than on the adoption willingness of doctors at tertiary hospitals. According to pathways S1a and S2a, a lack of convenience factors did not affect the strong adoption willingness of doctors at tertiary hospitals. This finding indicates that even when the marginal condition of the convenience factor is missing, these doctors still have a positive adoption willingness for AI-CDSSs. In contrast, looking at pathway N2b, convenience factors are a necessary condition for doctors at primary/secondary hospitals, and these doctors will only favor adoption of AI-CDSSs when convenience factors are present. Thus, convenience factors are the objective material factors influencing the willingness of these doctors to adopt AI-CDSSs. As tertiary hospitals are generally regional hospitals that have comprehensive hospital facilities and advanced information systems, such convenience factors are present; thus, they do not significantly influence the adoption of AI-CDSSs among doctors at these facilities. Unlike tertiary hospitals, primary/secondary hospitals are often county hospitals or primary health care institutions, with some located in remote rural areas and having fewer hardware and software resources to support their doctors. Therefore, the impact of convenience factors on the adoption willingness of doctors at these medical institutions is greater. The survey results imply that only when there is sufficient external support for doctors at these primary and secondary institutions will they actively adopt AI-CDSSs.

### Robustness Test

To test the robustness of our results, we adjusted the consistency threshold from 0.8 to 0.85 and 0.72 [44]. There were no substantial changes observed in the configuration of the pathways or parameters. The results indicated that the adjusted structure remained consistent with the original structure and the pathways were the same as those before the adjustment. Therefore, the results remained robust.

## Discussion

### Principal Findings

We constructed a theoretical framework based on the UTAUT and TOE framework using configurational thinking and fsQCA to configure 6 conditional elements. We explored the multiple concurrent factors and causal complex mechanisms that influence the willingness to adopt AI-CDSSs among clinical doctors at different medical institutions in China from technological, organizational, and individual perspectives. Our results provide a theoretical basis for the further integration of AI-CDSSs into clinical applications. The following are our key results.

We found that the paths driving high AI-CDSS adoption willingness among clinical doctors in tertiary hospitals fell into 6 categories, which were summarized in 3 configurations: technology driven, individual driven, and technology-individual dual driven. The paths driving high AI-CDSS adoption willingness among clinical doctors at primary/secondary medical institutions fell into 3 categories, which were summarized in 2 configurations: technology-individual and organization-individual dual driven.

Comparing tertiary hospitals with primary/secondary medical institutions, we observed some commonalities and some differences in the paths driving doctors to a positive willingness to adopt AI-CDSSs. In terms of commonalities, AI technology and individual factors play dominant roles in doctors’ adoption willingness. The doctors indicated their beliefs that AI-CDSSs can provide efficient diagnostic support and improve the quality of medical services. Moreover, they indicated that they are willing to try new technologies. In terms of differences, convenience factors had a greater impact on doctors at primary/secondary medical institutions. These doctors would actively adopt AI-CDSSs only with sufficient external support.

By studying doctors from different levels of medical institutions in China and their adoption paths of AI-CDSSs, we found that resource availability may be an important factor influencing the adoption willingness of doctors at different medical institutions. For example, tertiary hospitals have greater access to resources, such as funding and technical support, compared with primary/secondary hospitals. Additionally, differences in organizational culture and management styles and values may impact doctors’ attitudes toward AI-CDSSs. The characteristics of patient populations served by different levels of medical institutions may also influence doctors’ perceptions of AI-CDSSs, as patient needs and complexities can vary across hospital settings.

Finally, our research results can serve as a reference for other developing countries for promoting the application of AI-CDSSs or AI technologies in clinical treatment, contributing to enhancing the medical service capabilities of medical institutions.

### Theoretical Contributions

Our study contributes to the literature in several ways. First, we built a comprehensive analytical framework for doctors’ willingness to adopt AI technology. This is based on the UTAUT and TOE framework combined with specific characteristics and application scenarios for AI technology, with a focus on technical, organizational, and individual factors. Previous studies have focused mainly on the causal relationships between individual variables and the willingness to adopt AI technology. However, our study introduces multiple factors that can influence doctors’ willingness to adopt AI technology, namely, performance expectancy, perceived risks, convenience factors, social influence, technology anxiety, and personal innovativeness. Building on the UTAUT model, we incorporated additional factors, specifically personal innovativeness, technology anxiety, and perceived risks, into the analytical framework. As such, we were able to enrich the theoretical research regarding the factors influencing doctors’ willingness to adopt AI technology.

Second, we explored the synergistic effects influencing doctors’ willingness to adopt AI-CDSSs from a configuration perspective, expanding the application of the UTAUT model in explaining causal complexity. Although the UTAUT model is widely used to explain individual adoption of new technologies within organizations, existing studies on AI adoption have largely overlooked the complexity of causal relationships. Owing to limitations in research methods, existing technology adoption models have been unable to test and explain the impact of multiple conditions on doctors’ willingness to adopt AI-CDSSs. In our study, we empirically investigated the synergistic effects of 6 specific factors related to technology, organization, and individual aspects on doctors’ willingness to adopt AI-CDSSs from a configuration perspective. By addressing the aforementioned issues, we expand the application of the UTAUT model in explaining causal complexity.

Finally, we used the fsQCA method to analyze the configurations of doctors’ willingness to adopt AI-CDSSs at different medical institutions. Our results indicate that across different medical institutions, performance expectancy and personal innovativeness are the 2 important conditions for doctors to engender strong adoption willingness for AI-CDSSs, whereas perceived risks hinder adoption. Social influence can either promote or hinder doctors’ willingness to adopt AI-CDSSs, and convenience factors have a greater impact on doctors’ adoption willingness at primary/secondary medical institutions. In summary, our research extends the literature on doctors’ willingness to adopt AI-CDSSs and provides theoretical support for future practical applications.

### Practical Implications

From the perspective of the 6 configurations among the doctors at tertiary hospitals and the 3 configurations among the doctors at primary/secondary medical institutions, performance expectancy and personal innovativeness were the 2 indispensable and core conditions in the pathways to achieving strong willingness to adopt AI-CDSSs. Thus, AI product providers and health care managers should look closely at these factors when designing and implementing such systems. The organizational factor of facilitating conditions for doctors at primary/secondary medical institutions also appeared to be a necessary condition influencing the adoption willingness of doctors at these institutions. As such, we recommend the following measures for the AI-CDSS process.

### Importance of Performance Expectancy in Adoption

For AI product providers, improvements in the quality and applicability of AI products can be achieved by focusing on the designing of AI-CDSSs that meet the specific needs of different clinical doctors. This means involving these doctors in the design and development process of AI-CDSSs to ensure that these systems address practical needs. In addition, to ensure that AI-CDSSs comply with data privacy and security standards, security measures should be included to increase doctors’ trust in the systems. The systems should also be evaluated regularly for effectiveness and impact, and should be continuously improved based on feedback to ensure they are meeting user needs and expectations.

### Importance of Personal Innovativeness

Health care institution managers should provide the appropriate training and support for doctors before introducing AI-CDSSs. This can be done through various activities, such as videos and practical exercises, among others. Such training can help doctors become comfortable with using AI-CDSSs and increase their effectiveness, boosting doctors’ confidence in the systems and their willingness to adopt the systems. In addition, doctors should be actively encouraged to be innovative, cultivate innovation awareness, and improve their acceptance of new technologies.

### Importance of Addressing Technology Anxiety

Individual doctors need to actively participate in training to understand the basic functions and uses of AI-CDSSs. Some doctors may be skeptical of AI-CDSSs, fearing that these systems will replace their work or reduce work quality. Appropriate training can help change this negative mindset, encouraging doctors to recognize that these systems are meant to assist and enhance the efficiency and accuracy of clinical work, and not to replace doctors.

### Limitations

This study has some limitations that can serve as starting points for future research. First, our research scope was limited to medical institutions in mainland China, thereby lacking comparisons with doctors in foreign medical institutions regarding the adoption paths of AI-CDSSs. Second, differences between medical institutions at different levels, such as funding, staffing, and equipment configuration, may influence doctors’ perceptions, acceptance, and experience with AI-CDSSs, leading to variations in questionnaire responses. In addition, we used fsQCA to explore the driving mechanism underlying doctors’ adoption willingness based on the interactive matching of multiple conditions, which does not verify the impact of individual variables or a few factors on adoption willingness. To address the limitations of the fsQCA method, future research could consider including the use of structural equation modeling (SEM) to design a more complex model structure by considering the relationships between latent variables and the correlations between multiple observed indicators, thus providing a more comprehensive data analysis and explanation. Finally, the sample size of the questionnaire in primary/secondary hospitals was less than that in tertiary hospitals, but the hospitals included in this study were representative. Because AI has more value in areas with weak medical resources, our future research will focus on the willingness of primary hospitals to use AI. We will refine research plans, expand the sample size of the survey, and improve the compliance of the hospitals included.

### Conclusion

Our study built a comprehensive analytical framework for doctors’ willingness to adopt AI-CDSSs, considering specific characteristics and application scenarios for AI technology, with a focus on technical, organizational, and individual factors. We used fsQCA to explore doctors’ willingness to adopt AI-CDSSs in different types of medical institutions in China along with the factors influencing their willingness. From the perspectives of the 6 pathways of the doctors at tertiary hospitals and the 3 pathways of the doctors at primary/secondary hospitals, performance expectancy and personal innovativeness were 2 indispensable and core conditions in the pathways to achieving favorable willingness to adopt AI-CDSSs. The comparative analysis revealed both similarities and differences between doctors at tertiary hospitals and those at primary/secondary hospitals in terms of their adoption of AI-CDSSs. While technical and individual factors were found to be influential in driving adoption willingness across all hospitals, the impact of facilitating conditions differed between the different levels of medical institutions. Facilitating conditions were identified as a significant driver for adoption among doctors at primary/secondary hospitals, underscoring the importance of external support and resources in facilitating the adoption of AI-CDSSs in these settings.

In conclusion, the results of our research provide valuable insights into the factors influencing doctors’ willingness to adopt AI-CDSSs in different health care settings. By addressing performance expectancy, personal innovativeness, and organizational support, health care organizations can promote a more favorable environment for the implementation and utilization of AI technologies, ultimately enhancing clinical decision-making systems and improving patient care outcomes. Continued research and implementation of these strategies can further advance the integration of AI-CDSSs in health care and pave the way for the widespread application of AI technology in clinical practice.

By exploring the positive practice and policy promotion of AI-CDSS application in China, our research can provide a positive reference for the governments of developing countries with similar conditions and uneven distribution of medical resources. At the technical level, the effectiveness and safety of AI technology should be ensured, so that AI can meet the needs of clinical practice. At the organizational level, medical institutions should organize technical training, so that doctors can understand and learn the combination of AI technology and clinical practice, and build a new model of human-machine collaboration. For areas with relatively weak medical resources, the government and medical institutions should increase the infrastructure construction required for AI applications and provide adequate technical support to help doctors solve technical problems.

## Abbreviations

AI: artificial intelligence
AI-CDSS: artificial intelligence–driven clinical decision support system
CDSS: clinical decision support system
fsQCA: fuzzy set qualitative comparative analysis
TOE: technology-organization-environment
UTAUT: unified theory of acceptance and use of technology

## Data Availability

The raw data supporting the study results are available from the authors upon reasonable request.
